# Increased lactate dehydrogenase activity is dispensable in squamous carcinoma cells of origin

**DOI:** 10.1038/s41467-018-07857-9

**Published:** 2019-01-09

**Authors:** A. Flores, S. Sandoval-Gonzalez, R. Takahashi, A. Krall, L. Sathe, L. Wei, C. Radu, J. H. Joly, N. A. Graham, H. R. Christofk, W. E. Lowry

**Affiliations:** 1 Department of Molecular Cell and Developmental Biology, UCLA, Los Angeles, 90095 CA USA; 2 Broad Center for Regenerative Medicine, UCLA, Los Angeles, 90095 CA USA; 3 Division of Dermatology, David Geffen School of Medicine, UCLA, Los Angeles, 90095 CA USA; 4 Department of Biological Chemistry, UCLA, Los Angeles, 90095 CA USA; 5 Department of Pharmacology, UCLA, Los Angeles, 90095 CA USA; 6 Department of Engineering, USC, Los Angeles, 90089 CA USA; 7 Mork Family Department of Chemical Engineering and Materials Science, University of Southern California, Los Angeles, 90089 CA USA; 8 Norris Comprehensive Cancer Center, University of Southern California, Los Angeles, 90089 CA USA; 9 Molecular Biology Institute, UCLA, Los Angeles, 90095 CA USA; 10 Jonsson Comprehensive Cancer Center, UCLA, Los Angeles, 90095 CA USA

## Abstract

Although numerous therapeutic strategies have attempted to target aerobic glycolysis to inhibit tumor progression, these approaches have not resulted in effective clinical outcomes. Murine squamous cell carcinoma (SCC) can be initiated by hair follicle stem cells (HFSCs). HFSCs utilize aerobic glycolysis, and the activity of lactate dehydrogenase (Ldh) is essential for HFSC activation. We sought to determine whether Ldh activity in SCC is critical for tumorigenesis or simply a marker of the cell type of origin. Genetic abrogation or induction of Ldh activity in HFSC-mediated tumorigenesis shows no effect on tumorigenesis as measured by number, time to formation, proliferation, volume, epithelial to mesenchymal transition, gene expression, or immune response. *Ldha*-null tumors show dramatically reduced levels of glycolytic metabolites by metabolomics, and significantly reduced glucose uptake by FDG-PET live animal imaging. These results suggest that squamous cancer cells of origin do not require increased glycolytic activity to generate cancers.

## Abstract (web summary)

Most tumours are characterized by increased aerobic glycolytic activity. Here the authors show that elevated  aerobic glycolysis is not essential for cancer initiation by testing the effect of lactate dehydrogenase depletion on the ability of hair follicle stem cells (HFSCs) to form squamous cell carcinoma (SCC) in mouse genetic models.

## Introduction

Most tumors are characterized by increased glucose uptake and lactate production, a phenomenon known as the Warburg effect or aerobic glycolysis. Elevated glucose uptake and glycolysis can power the production of essential metabolites and cell products required for proliferation^1–3^. Aerobic glycolysis culminates in the NADH-dependent reduction of pyruvate to lactate by lactate dehydrogenase (Ldh)^3–6^. Although lactate was once considered a waste product of glycolysis, it has been argued that lactate production may be a primary purpose of the Warburg effect, as lactate impacts angiogenesis, immune response, acidification of the microenvironment, motility of cancer cells, and the regeneration of NAD^+^^5,7,8^. Moreover, inhibition of Ldh activity, which reduces lactate production, has been shown to impair the growth of tumor cells in vitro^9,10^. Regardless, despite decades of research in this area, it is still not clear whether the increased conversion of glucose to lactate is necessary for tumor initiation or progression, or just a by-product of altered metabolism.

In fact, while there is a mountain of data suggesting that lactate dehydrogenase activity is important for cancer cell growth in in vitro and ex vivo models^11–14^, the relevance of lactate production to tumor initiation and progression in vivo has not been well explored. One study that used a model of lung carcinoma driven by oncogenic *Ras* coupled with deletion of *Ldha* showed a regression of tumors, suggesting a requirement of Ldh activity for maintenance of tumor cells^11^. Ldh activity was abrogated in the entire tissue in that model, however, which left uncertain the role of glycolytic activity specifically in cancer cells of origin.

Here, we use a model of cutaneous squamous cell carcinoma (SCC) to model tumor formation and progression from hair follicle stem cells (HFSCs). HFSCs, which have been shown to be cancer cells of origin for squamous cell carcinoma^15,16^, exhibit a high level of glycolytic activity during homeostatic conditions^17^. We therefore sought to determine whether aerobic glycolysis is required for SCC progression or whether SCC tumors are glycolytic simply because they were initiated by glycolytic HFSCs.

## Results

### Induction of glycolysis in a genetic model of squamous cell carcinoma

To investigate the relevance of lactate production to cutaneous squamous cell carcinoma, we used a previously demonstrated murine model of SCC driven by gain of oncogenic *Ras* coupled with loss of *p53* activity in HFSCs^15^. Transgene expression was targeted to HFSCs using transgenic alleles such as *K15CrePR* or *Lgr5CreER*^18,19^ with induction of Cre recombination through administration of mifepristone or tamoxifen.

**Fig. 1:**
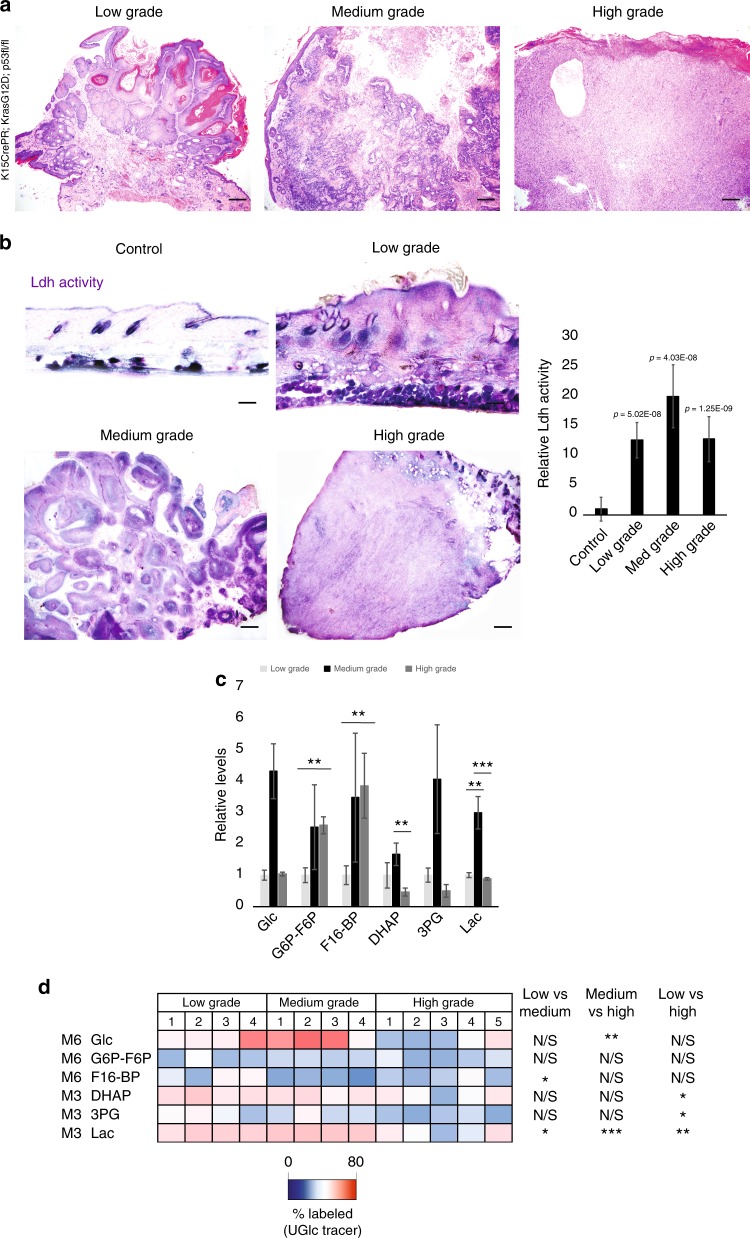
Correlation of Ldh activity and tumorigenesis of SCC. Haematoxylin and eosin staining showing histology of squamous tumors of various stages: low-grade papilloma/keratoacanthoma stage, medium-grade squamous carcinoma, and high-grade undifferentiated squamous carcinoma. Scale bars, 200 µm. **b** In situ Ldh activity assay highlights maximal potential Ldh activity in murine skin during *Kras*G12D/*p53* mediated SCC formation from HFSCs. Purple stain indicates relative Ldh activity in the skin of control (top left; +mifepristone/-oncogenic stimulation) versus hyperplasia stage (top right; +mifepristone/+oncogenic stimulation), papilloma stage (lower left), and high-grade SCC (lower right) Scale bars, 200 µm. **b** (right panel) Ldh activity in lysate from tissue of the indicated stage of tumorigenesis. Each bar represents the average activity for each tumor type where *n* = 6 tumors per stage from 24 animals. Shown as mean ± SEM. Paired *t* test was performed, *p* < 0.05 shown for each tumor type versus control tissue. **c** Metabolites were extracted and analyzed by LCMS from four tumors (1–4) from each stage of tumorigenesis (12 animals total). Glucose (Glc) and glycolytic intermediate levels are shown as relative values to the low grade tumor group. **d** following intraperitoneal injection of animals with [U-^13^C_6_]glucose, tumors at various stages were isolated and profiled by metabolomics. Labeled metabolites were extracted and analyzed by LC–MS from four tumors (1–4) from each stage of tumorigenesis (12 animals total). Heatmap depicts percentage of indicated isotopomers of glucose and glycolytic intermediates labeled from the U-13C-glucose tracer. For (**c**), error bars denote SD (*n* = 4 individual tumors). For (**c**, **d**), *P<0.05; **P<0.01; ***P<0.001 by Student’s *t* test

We first investigated whether lactate synthesis correlates with tumor grade. We typically characterize squamous tumors in the following categories: hyperplasia, low grade papilloma/keratoacanthoma stage, medium-grade squamous carcinoma, and high-grade undifferentiated squamous carcinoma (Fig. 1a). While Ldh activity, as measured by in situ activity, was elevated relative to normal skin in all examined tumor grades, the activity of Ldh appeared to peak at the papilloma/keratoacanthoma stage, and decreased somewhat in high-grade tumors (Fig. 1b). These results were confirmed by a more quantitative analysis of Ldh activity in lysate from isolated tumor tissues of various stages (Fig. 1b).

For a more comprehensive metabolic analysis, we also performed liquid chromatography mass spectrometry (LCMS)-based metabolomics on tumor tissue from various stages of tumorigenesis. This analysis showed that as the skin became filled with tumorigenic cells, the steady-state levels of some glycolytic metabolites increased, particularly in the medium grade stage (Fig. 1c). We also performed glucose tracing experiments in which animals with tumors at various stages were intraperitoneally injected with [U-^13^C_6_] glucose 15 min prior to skin and tumor harvesting and metabolite extraction in order to follow the conversion of glucose to various metabolites. More ^13^C-labeled lactate in the M3 isotopomer form was detected in the medium- grade papillomas than in the low-grade or high-grade tumors (Fig. 1d). These analyses are consistent with an increased conversion of glucose to lactate as SCC initiated and progressed, with a peak of lactate labeling from glucose in the medium grade, and a drop off somewhat in high-grade tumors.

Finally, we measured gene expression changes in HFSCs as they proceed through various stages of tumorigenesis. Expression of *Ldha* in HFSC-derived tumors was not elevated relative to normal HFSCs (Supplementary Figure 1), which we previously showed to have high expression of *Ldha* relative to other types of cells in the skin^17^. In contrast, expression of other glycolytic enzymes, such as *Pgm, HK, Pgk, Pkm2*, and *Eno*, were further increased during tumorigenesis. Furthermore, transcriptome data demonstrated that transporters for lactate, pyruvate, glucose, and glutamine were all upregulated. These data suggested that despite the relatively high glycolytic rate of HFSCs under homeostatic conditions^17^, glycolysis may be further induced upon tumorigenesis.

### Genetic abrogation of Ldh activity during initiation of SCC

To determine whether induction of lactate production in HFSCs is required for squamous cell carcinoma initiation, we induced tumorigenesis in HFSCs in the context of *Ldha* deletion. For this purpose, we coupled conditional deletion of *p53* and activation of constitutively active *Kras* (LSL-*Kras*G12D) with inducible deletion of floxed-*Ldha* with either *K15CrePR*- or *Lgr5CreER*-mediated recombination, which we previously showed effectively abrogates lactate production in HFSCs^15,17,20^.

**Fig. 2:**
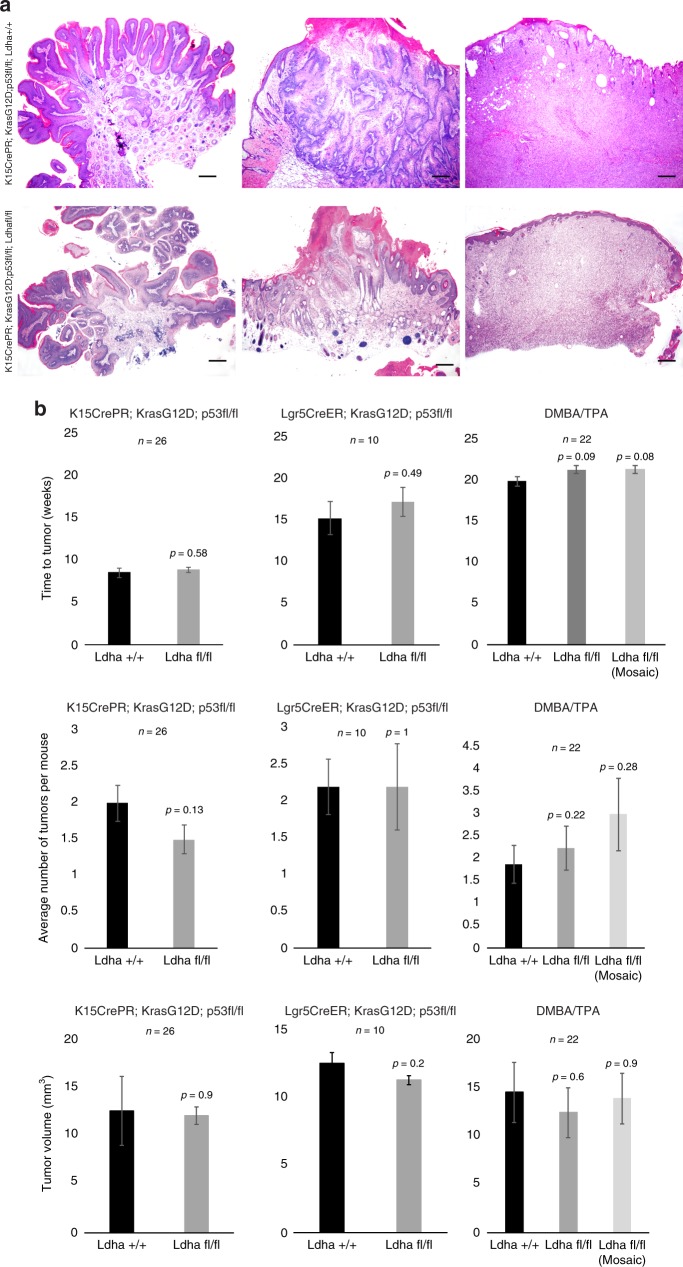
Loss of Ldha does not affect tumor initiation, progression or pathology. **a** Pathology of *Kras*G12D/*p53* induced tumors from HFSCs with and without *Ldha* deletion appear indistinguishable across tumorigenesis. Scale bars, 200 µm. **b** Quantification of time to tumor formation (left), the number of tumors formed, or the tumor volume showed no significant difference between *Ldha* expressing vs. *Ldha* deleted tumors. Also shown is results from animals where *Kras*G12D/*p53* was generated under the control of the *Lgr5CreER*, or in independent experiments where chemical carcinogenesis (DMBA/TPA) was carried out. Each bar represents the average across indicated values for *n*. Shown as mean ± SEM. Paired *t* test was performed, *P* < 0.05 shown for each genotype versus wild-type control

Contrary to numerous observations linking glycolysis and lactate production to tumorigenesis, *Kras*G12D-*p53*fl/fl-mediated SCC tumor formation from HFSCs was not affected by loss of *Ldha* (Fig. 2a). Quantification of tumorigenesis showed that neither the timing, volume, nor number of tumors formed was affected by loss of *Ldha* (Fig. 2b). These results were confirmed by induction of oncogenesis by a distinct HFSC-specific Cre (*Lgr5CreER*) (Fig. 2b) and through DMBA/TPA administration, a classical SCC paradigm (Fig. 2b)^21^. DMBA/TPA tumors were stratified as null or mosaic for *Ldha* expression.

We next attempted to more carefully characterize tumors generated from wild-type or *Ldha*-null HFSCs. *Ldha* wild-type and null tumors were not distinguishable pathologically; both sets of tumors showed hallmarks of spindle cell proliferation, papillomatous papulae, infiltration, etc (Supplementary Figure 2a). Because it has been suggested that tumor-generated lactate can suppress immune activity, we additionally explored the possibility that tumors could elicit distinct immune responses depending on their expression of *Ldha* (Supplementary Figure 2b and c). However, we were unable to detect a distinct pattern of immune response in the absence of Ldh activity. We also compared proliferation, epithelial to mesenchymal transition (EMT), markers of HFSC fate, and total gene expression (Supplementary Figure 3a). Staining for Ki67 to measure proliferation did not identify any significant difference between tumors that express or lack *Ldha*. Staining for markers of EMT showed that all tumors upregulated mesenchymal markers (such as fibronectin and tenascin C) and downregulated epithelial markers (such as keratin 14), regardless of *Ldha* status. HFSCs are known to express markers such as CD34 and Sox9 (as well as secrete Tnc)^22–24^. Immunostaining for these HFSC markers showed that tumors generated from HFSCs do indeed continue to express *Sox9* and *CD34*, and that this is not affected by loss of *Ldha*. Hypoxia is a common phenotype in a majority of malignant tumors and has been shown to alter tumor metabolism, vascularization, and epithelial-to-mesenchymal transition. There were no observed differences in hypoxia levels in tumors due to loss of *Ldha* (Supplementary Figure 3b). Finally, to identify any molecular changes in tumors due to loss of *Ldha*, we performed RNA-seq on six tumors that express *Ldha* versus six that were deleted for *Ldha*. Following normalization, stringent analysis failed to detect significant gene expression changes associated with loss of *Ldha*. In particular, we examined the same genes described to be induced during SCC generation from HFSCs (Supplementary Figure 1), and found that none of these genes or pathways were distinctly different in tumors formed in the absence of Ldh activity (Supplementary Figure 3c). Collectively, these data suggest that *Ldha* is not essential for SCC tumor formation.

### Measuring metabolism in *Ldha*-null tumors

**Fig. 3:**
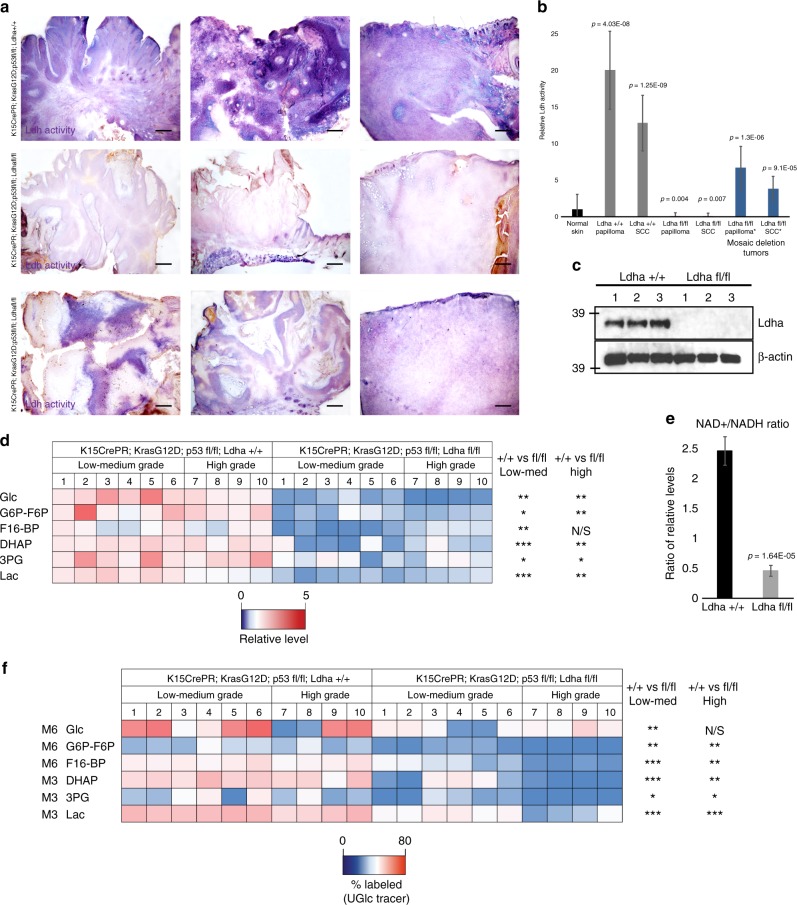
Metabolic effects of loss of Ldha during tumorigenesis. **a** In situ measurement of maximal Ldh activity in tumors with and without *Ldha* deletion shows a dramatic loss of activity in most tumors from the *Ldha* deletion mice (middle row). Activity is indicated by purple color; pink is a nuclear counterstain. In addition, some tumors formed in deletion mice show mosaicism for Ldh activity, presumably due to mosaic deletion of *Ldha* mediated by CrePR induced recombination. Scale bars, 200 µm. **b** Ldh activity in lysate derived from tumors of the indicated stages. Those samples with * indicated tumors that were deemed to be mosaic for *Ldha* deletion by the in situ assay. Each bar represents the average signal for each tumor type where *n* = 6 mice per tumor stage and genotype from 42 animals. Shown as mean ± SEM. Paired *t* test was performed, *P* < 0.05 shown for each tumor type versus control skin. **c** Western blotting for *Ldha* protein indicated the effectiveness of the genetic deletion. **d** Heatmap depicts relative levels of glycolytic intermediates as measured by LCMS in tumors from animals with and without *Ldha* expression. Each column represents metabolite measurements from an individual animal, and 20 animals were used, 10 of each genotype. **e** NAD^+^/NADH ratio in *Ldha* wild-type (+/+) vs. *Ldha* null (fl/fl) tumors. Each bar represents the relative NAD^+^/NADH ratio where *n* = 10 mice per genotype. Shown as mean ± SEM. Paired *t* test was performed, *P* < 0.05 shown for knockout versus wild-type tumors. **f** Heatmap depicts percentage of glycolytic intermediate isotopomers in tumors with indicated genotypes from animals IP injected with [U-^13^C_6_] glucose 15 min prior to tumor harvesting. For (**d**, **f**), ^∗^*P* < 0.05; ^∗∗^*P* < 0.01; ^∗∗∗^*P* < 0.001; NS, *P* > 0.05. Student’s paired *t* test

One explanation for the lack of effect of loss of *Ldha* on tumor formation could be that another Ldh isoform (*Ldhb, Ldhc*, or *Ldhd*) was able to compensate for the loss. To determine whether *Ldha*-null tumors had indeed lost Ldh enzymatic activity, we assayed activity with several independent methods. First, we used an in situ activity assay to identify tumors or areas within tumors that show Ldh activity. All tumors genotypically positive for *Ldha* showed robust Ldh activity (Fig. 3a). Amongst the *Ldha*-null tumors, most had completely lost Ldh activity in this assay, while some showed mosaic activity, presumably due to the inducible method of Cre recombination employed. There was no difference in appearance between the tumors that had full activity, lacked activity, or were mosaic for Ldh activity (Fig. 3a). Second, we examined Ldh activity in cell lysate from isolated tumors, which confirmed the absence of Ldh activity in *Ldha*-null tumors (Fig. 3b, c). Finally, we used LCMS-based metabolomics to measure the relative level of lactate in all the tumors analyzed. This analysis showed that lactate and most of the other glycolytic metabolites were dramatically lower in *Ldha*-null tumors (Fig. 3d). Relative levels of TCA cycle metabolite pools and glucose-labeled TCA metabolites were also analyzed in *Ldha* wild-type versus *Ldha*-null tumors and not many significant changes were observed (Supplementary Figure 4). In addition, the NAD^+^/NADH ratio was reduced in *Ldha*-null tumors, consistent with decreased oxidation of NADH to NAD^+^ (Fig. 3e). Further, animals were injected with [U-^13^C_6_] glucose prior to tumor harvesting to measure glucose utilization. Glucose labeling of lactate as well as all other examined glycolytic metabolites was dramatically reduced in *Ldha*-null tumors (Fig. 3f). Together, these data strongly suggest that the tumors formed in genotypically *Ldha*-null mice in fact lost Ldh activity without compensation from any other Ldh isoform (b, c, d).

**Fig. 4:**
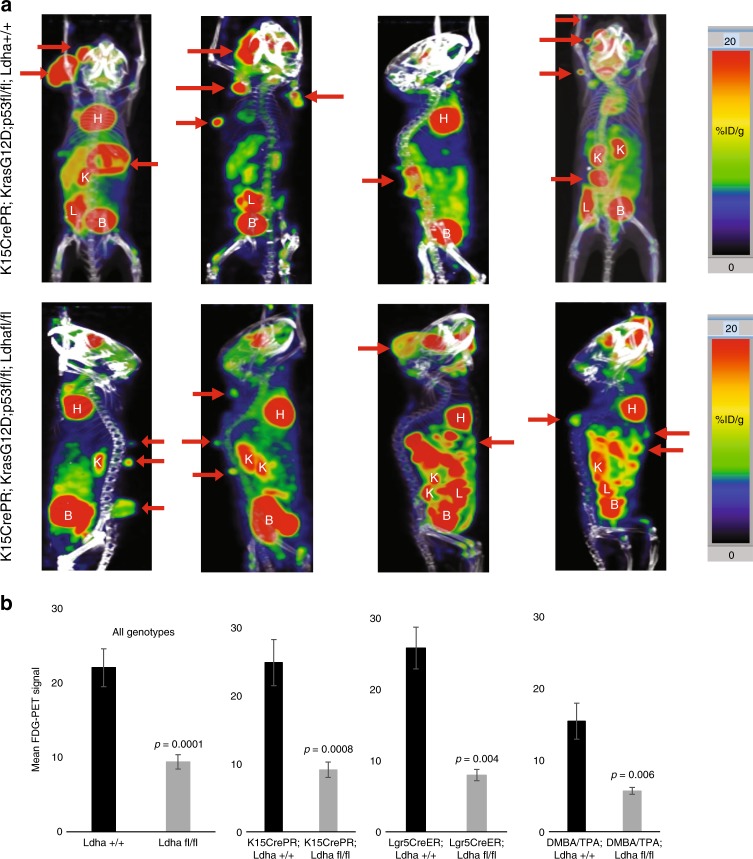
Absence of Ldha leads to decreased Glucose uptake in tumors. **a** Positron Emission Tomography imaging after injection of FDG, a glucose analogue was used to demonstrate the relative degree of glucose uptake across tumors formed in the indicated genotypes. Red coloration indicates a high level of glucose uptake, and further demonstrates that SCC tumors are highly glycolytic. Red arrows indicate tumors. H = heart, K = kidney, L = liver, B = bladder. **b** Quantification of FDG-PET signal showed that *Ldha* null tumors (from either *K15CrePR*, *Lgr5CreER*, or DMBA/TPA mice) consistently show lower glucose uptake. Each bar represents the mean FDG-PET signal where *n* = 6 mice per genotype. Shown as mean ± SEM. Student’s paired *t* test was performed, *P* < 0.05 shown for knockout versus wild-type tumors

To determine whether the depletion of glycolytic intermediates in *Ldha*-null tumors (Fig. 3d) was due to reduced glucose consumption, we performed FDG-PET on our mice with *Ldha* wild-type and null skin tumors. FDG is a glucose analogue that can be non-invasively imaged by positron emission tomography (PET)^25,26^. Tumors are known to be highly glycolytic and take up significantly more FDG than normal tissue, and this was observed in all tumors expressing *Ldha* (Fig. 4a). On the other hand, tumors null for *Ldha*, showed significantly less FDG uptake relative to *Ldha* wild-type tumors. This was the case in both tumors induced by *Kras*G12D/loss of *p53*, as well as tumors generated by DMBA/TPA (Fig. 4b). These data indicate that tumors deficient in Ldh activity abrogate their glucose uptake.

### Abgrogation of Ldh activity during tumor progression

**Fig. 5:**
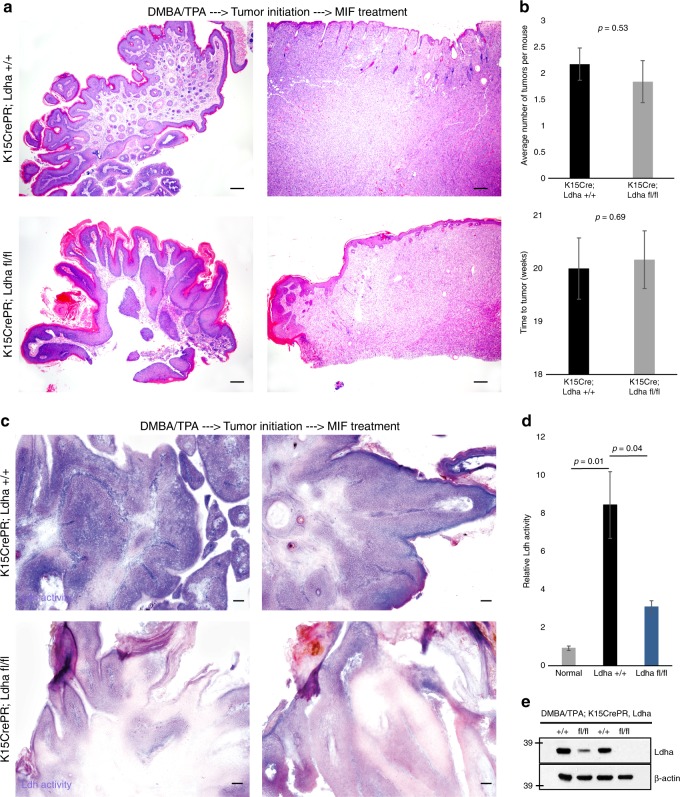
Deletion of Ldh activity in nascent tumors does not affect tumor progression. **a** Taking advantage of chemical carcinogenesis coupled with transgenic deletion of *Ldha*, we allowed tumor initiation to begin and then deleted *Ldha* by administration of Mifepristone in transgenic animals. Haematoxylin and Eosin stain shows similar histology in wild-type and Lda-null tumors. Scale bars, 200 µm. **b** Quantification of tumor formation and progression failed to uncover an effect of loss of *Ldha*. Each bar represents the mean where *n* = 12 mice per genotype. Shown as mean ± SEM. Paired *t* test was performed, *p* < 0.05 shown for knockout versus wild-type tumors. **c** In situ Ldh activity assay confirmed that deletion of *Ldha* after tumorigenesis had begun was effective at abrogating Ldh activity in tumors. Activity is indicated by purple color; pink is a nuclear counterstain. Scale bars, 100 µm. **d** A plate reader assay for Ldh activity also showed that the *Ldha* deletion was effective. Each bar represents the relative ldh activity signal for each genotype type, where *n* = 6 mice per genotype. Shown as mean ± SEM. Paired *t* test was performed, *p* < 0.05 shown for each tumor genotype vs. control skin. **e** Western blotting for *Ldha* and a loading control to show the effectiveness of *Ldha* deletion after tumor initiation

The fact that loss of Ldh activity did not affect tumor initiation raised the possibility that lactate production is important for tumor progression as opposed to tumor initiation. To test this hypothesis, we administered DMBA/TPA for several weeks until the first signs of tumorigenesis^27–29^ then deleted *Ldha* by Cre activation with Mifepristone in transgenic animals. The experiment was allowed to continue for several more weeks, and the results were quantified. We were unable to detect an effect of loss of Ldh activity even in existing tumors (Fig. 5a). Deletion of *Ldha* in the midst of tumor formation did not affect the timing or degree of tumorigenesis (Fig. 5b). *Ldha* deletion was confirmed by in situ staining for Ldh (Fig. 5c), Ldh activity in tumor lysate (Fig. 5d), and western blotting (Fig. 5e).

### Stimulation of Ldh activity during tumorigenesis

**Fig. 6:**
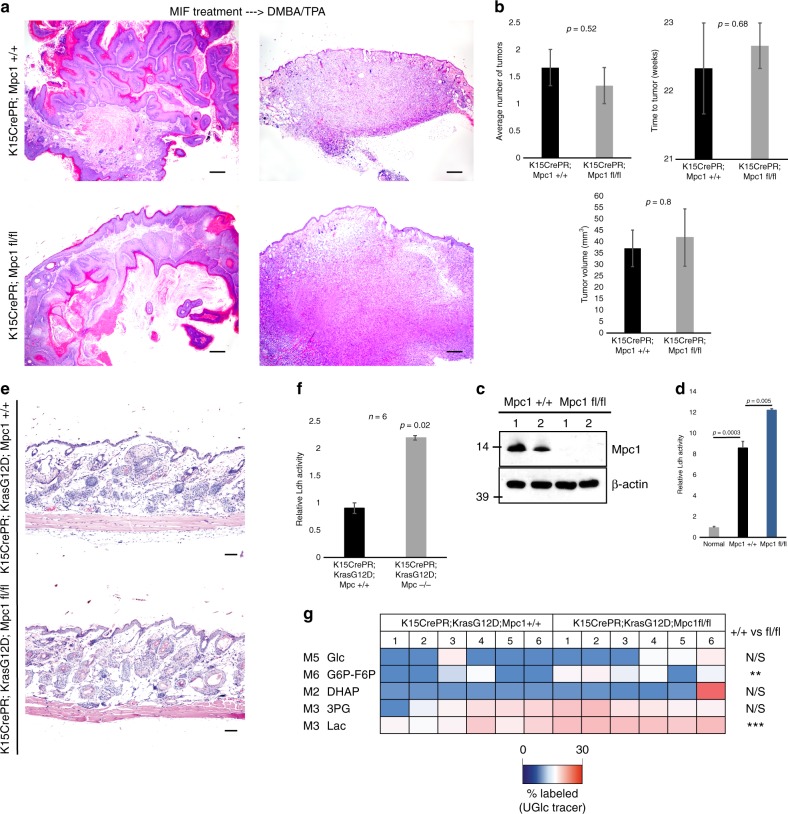
Induction of Ldh activity by deletion of mitochondrial pyruvate transport does not affect tumor initiation or progression in SCC. **a** Coupling DMBA/TPA carcinogenesis to transgenic deletion of mitochondrial pyruvate carrier function allowed for an examination of tumorigenesis following stimulation of Ldh activity. Haematoxylin and eosin stain shows similar histology in wild-type and *Mpc1*-null tumors. Scale bars, 200 µm. **b** Quantification of time to tumor formation, number of tumors, and tumor volume formed showed that *Mpc1* deletion did not affect tumorigenesis. Each bar represents *n* = 12 mice per genotype. Shown as mean ± SEM. Paired *t* test was performed, *P* < 0.05 shown for knockout vs. wild-type tumors. **c** Western blotting indicated that the genetic deletion of *Mpc1* was effective. **d** Ldh activity on lysate from normal skin and wild-type vs. *Mpc1*-null tumors. Each bar represents the relative Ldh activity signal for each genotype type where *n* = 12 mice per genotype. Shown as mean ± SEM. Paired *t* test was performed, *P* < 0.05 shown for each tumor genotype vs. control skin. **e** Mice with just gain of *Kras*G12D were crossed with floxed-*Mpc1* mice to generate one-hit mice with and without *Mpc1* expression. Haematoxylin and eosin stain shows similar hyperplasia in wild-type and *Mpc1*-null tumors. Scale bars, 100 µm. **f** *Ldha* activity in lysate generated from wild-type vs. *Mpc1*-null tumors. Each bar represents the relative Ldh activity signal for each genotype type, where *n* = 3 mice per genotype. Shown as mean ± SEM. Paired *t* test was performed, *P* < 0.05 shown for wild-type vs. knockout hyperplastic skin. **g** [U-^13^C_6_]glucose tracing for glycolytic intermediates shows that while glucose uptake did not change, the production of lactate was increased by loss of *Mpc1* in HFSCs. Labeled metabolites were extracted and analyzed by LC–MS from hyperplastic tissue from each genotype. Heatmap depicts percent labeled glycolytic intermediate isotopomers from tissue isolated from six mice per genotype. Student’s paired *t* test was performed, ^∗^*P* < 0.05; ^∗∗^*P* < 0.01; ^∗∗∗^*P* < 0.001; NS, *P* > 0.05; *n* = 12

To complement the *Ldha* deletion experiments, we examined whether enhancing Ldh activity influences SCC tumorigenesis. For this purpose, we genetically induced lactate production through the deletion of mitochondrial pyruvate carrier (*Mpc1*), an obligate component of the mitochondrial pyruvate carrier. We and others previously showed that inhibiting the ability of pyruvate to enter the mitochondria leads to increased Ldh activity^17,30^, and deleting *Mpc1* with *K15CrePR* or *Lgr5CreER*-mediated recombination effectively increases lactate production specifically in HFSCs^17^. Here, we used a floxed *Mpc1* allele in conjunction with DMBA/TPA to induce tumorigenesis in cells with enhanced Ldh activity. Deletion of *Mpc1* in these tumors led to a two-fold increase in Ldh activity, (Fig. 6c, d). However, as with Ldh deletion, Ldh activation failed to affect the timing or degree of tumorigenesis (Fig. 6a, b). We also considered the possibility that the combination of *Kras*G12D and *p53* loss may be  too  potent  for *Mpc1* loss to further increase tumorigenesis. To address this possibility, animals were created that coupled *Mpc1* loss to the single oncogenic hit of *Kras*G12D (Fig. 6e), which, when induced in HFSCs, only leads to benign hyperplasia in the follicle^15^. However, although Ldh activity (Fig. 6f) and lactate labeling by glucose carbons (Fig. 6g) were elevated by *Mpc1* loss, the rate or degree of tumorigenesis was unaltered in this model (Fig. 6a, b).

### Examination of metabolite flux in the absence of Ldh activity

**Fig. 7:**
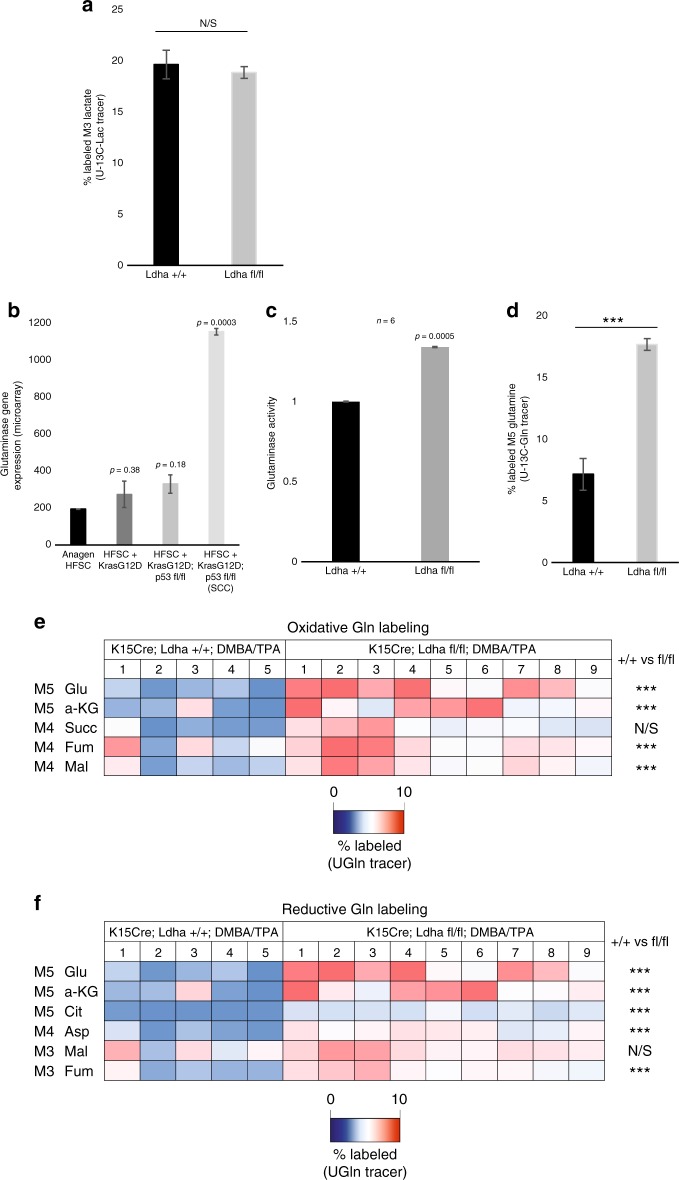
Glutamine uptake and metabolism are elevated Ldha-null tumors. **a** Percentage of M3 lactate in *Ldha* +/+ vs. fl/fl tumors from mice injected with [U-^13^C_3_]lactate 15 min prior to tumor harvesting and metabolite extraction. Student’s paired *t* test was performed, ^∗^*P* < 0.05; ^∗∗^*P* < 0.01; ^∗∗∗^*P* < 0.001; NS, not significant; *n* = 12. **b** Glutaminase mRNA levels in HFSCs at various stages of tumorigenesis. Each bar represents *n* = 3 mice per condition. Shown as mean ± SEM. Student’s paired *t* test is shown for each condition vs. anagen HFSCs. **c** Glutaminase activity in lysate from wild-type and *Ldha*-null tumors. Each bar represents the relative glutaminase activity signal for each genotype type, where *n* = 3 mice per genotype. Shown as mean ± SEM. Student’s paired *t* test was performed, *P* < 0.05 shown for wild-type vs. knockout tumors. **d** Percentage of M5 glutamine in *Ldha* +/+ vs. fl/fl tumors from mice injected with [U-^13^C_5_]glutamine 15 min prior to tumor harvesting and metabolite extraction. Student’s paired *t* test was performed, ^∗^*P* <  0.05; ^∗∗^*P* < 0.01; ^∗∗∗^*P* < 0.001; NS, not significant; *n* = 12. **e**–**f** Heatmaps depict percentage of TCA cycle intermediate isotopomers in oxidative and reductive glutamine metabolism, respectively, in tumors with the indicated genotypes. Animals were IP injected with [U-^13^C_5_] glutamine 15 min prior to tumor harvesting. Student’s paired t-test was performed; ^∗^*P* < 0.05; ^∗∗^*P* < 0.01; ^∗∗∗^*P* < 0.001; NS, not significant; *n* = 14

These data suggest that aerobic glycolysis is not necessary for tumor initiation or progression. It is thought that the increased glucose uptake and lactate production characterized by the Warburg effect increases the availability of metabolic intermediates needed for macromolecule biosynthesis. We therefore examined whether *Ldha*-null tumors use alternative carbon sources to fuel biosynthetic processes. Recent studies have shown that lactate in the blood can readily be used as a carbon source to power the TCA cycle. Although lactate is thought to be metabolized primarily through Ldh-dependent pyruvate generation, we considered whether *Ldha*-null tumors could oxidize imported lactate via alternative dehydrogenases to power the TCA cycle. To test this possibility, we injected mice with [U-^13^C_3_] lactate and performed metabolomics on both wild-type and *Ldha*-null tumors. However, there was no significant difference in the amount of lactate uptake in the absence of Ldh activity (Fig. 7a). While metabolomic analyses showed lactate lactate-labeling of TCA cycle intermediates including citrate, alpha-ketoglutarate, succinate, fumarate, and malate, though there was no significant difference in labeling between wild-type or *Ldha*-null tumors (Supplementary Figure 5).

Finally, we examined whether *Ldha*-null tumor cells increase glutamine utilization from the environment as a carbon source. Glutamine is imported into the cell through the Slc1a5 transporter and can fuel the TCA cycle through glutaminase-mediated conversion to glutamate. mRNA levels of both *Slc1a5* and glutaminase were upregulated in HFSC-induced SCC (Fig S1 and Fig. 7b), raising the possibility that glutamine metabolism may be increased in SCC formation^35^. To determine whether loss of Ldh activity promotes glutamine metabolism, we measured glutaminase activity in tumor lysate. *Ldha*-null tumors exhibited elevated glutaminase activity relative to wild-type tumors (*n* = 6) (Fig. 7c). In addition, tumor glutamine metabolism was assessed through tumor glutamine tracing with injected [U-^13^C_5_] glutamine. Metabolomics analysis of glutamine-labeled tumors indicated that *Ldha*-null tumors did indeed take up more glutamine than wild-type tumors (Fig. 7d). Moreover, *Ldha*-null tumors showed increased glutamine labeling of several TCA cycle metabolites via oxidative glutamine metabolism (Fig. 7e) and reductive glutamine metabolism (Fig. 7f), consistent with increased use of glutamine as a biosynthetic carbon source in the absence of Ldh activity.

## Discussion

Based on decades of research showing that nearly all tumors display increased lactate production, our null hypothesis was that deletion of *Ldha* would block tumor formation from HFSCs. In addition, *Ldha* deletion in a model of lung tumor formation caused tumors to regress^11^. Despite the fact that this lung model also used *Kras*G12D and floxed *p53*, the outcome was different than what was observed here in a model of cutaneous SCC. The difference could be due to performing the experiments in distinct tissues, but the lung study also used deletion of *Ldha* in the entire tissue, and deleted *Ldha* only after tumors were established^11^. Altered metabolism of cells in the lung tumor environment due to global *Ldha* deletion may have resulted in a different outcome. Alternatively, the lung and the epidermis are distinct tumor environments with distinct nutrient availabilities. The tumor environment may enable cutaneous SCC to adapt to nutrients generated by other cells of the epidermis, whereas alternative nutrient limitation may necessitate lung cancer dependence on glucose metabolism. In fact, a recent study showed that only certain types of lung tumors are sensitive to inhibition of glycolysis and certain lung tumors require inhibition of both glycolysis and glutamine pathways to block tumorigenesis^5^, suggesting that different types of tumors have different metabolic requirements. In addition, Ldh expression appears to be distinct between the lung and skin. In the lung, it appeared from the Seth et al. study that *Ldhb* is expressed significantly higher than *Ldha*, while the reverse is true in the skin^11,17^ (Fig. 1). It is interesting that after deletion of *Ldha*, cutaneous SCC still formed from HFSCs without compensation by *Ldhb*. Furthermore, deletion of *Ldha* in SCC appeared to strongly abrogate total Ldh activity, demonstrating that *Ldha* is the dominant isoform in the skin model.

Previous studies have shown *LDHA* expression predicts worse survival in clear cell renal cell carcinoma^21^, cholangiocarcinoma^30^, and breast cancer^31^. Taking advantage of multiple databases for human cancers and a novel aggregator called CANCERTOOL^32^, it is clear that *LDHA* expression is increased in a variety of human cancers (Supplementary Figure 6a). However, despite this increase in expression (and presumably activity), *LDHA* expression levels do not universally correlate with patient outcomes (Supplementary Figure 6b). The fact that *LDHA* expression does not always predict survival even in human tumors in which it is upregulated is consistent with our findings that *Ldha* expression is elevated in SCC but not required for SCC tumor growth in the mouse skin and may underlie fundamental differences in metabolic dependencies or enhanced metabolic flexibility of squamous cell carcinomas relative to other tumor types.

In the current study, we found that deletion of *Ldha* neither before nor after tumor formation had an effect on the outcome, demonstrating that Ldh activity in cancer cells of origin is not required for tumor initiation or progression in SCC. These results are consistent with the notion that high Ldh activity in tumors could be due to the fact that at least some cancer cells of origin are high in Ldh activity. Indeed, if the Warburg nature of SCC is more a reflection of expansion of phenotype of the cell from which it arose (Supplementary Figure 3), this could explain why loss of Ldh activity had no significant effect on tumorigenesis. It is also possible that the increased Ldh activity observed in tumors could be oncogene or even tissue dependent, and additional studies are necessary to determine to what extent cell of origin, tumor suppressors, oncogenes, and tumor microenvironment contribute to this common phenotype.

The longer-term question is why do tumors produce so much lactate if it is not required for their initiation or maintenance? Lactate was previously considered simply a waste product of glycolysis, which could explain why loss of lactate production in SCC does not appear to have a consequence in our model. On the other hand, recent studies have indicated that lactate is potentially an important molecule to suppress the immune response to tumor formation, affect angiogenesis, acidify the microenvironment, and increase the motility of cancer cells. Additionally, the conversion of pyruvate to lactate by Ldh enzyme produces NAD^+^, and the NADH/NAD^+^ ratio is thought to be important in numerous oxidoreductase-based metabolic reactions (reviewed in refs. ^27–29^). As a result, some have argued that the entire purpose of the Warburg effect is to produce lactate for the sake of driving these events that are known to be important for tumor formation^27^.

In light of data showing that lactate can act at a distance, it is also important to consider where lactate is produced. Two studies in mouse and human demonstrated that in fact lactate is found at very high concentration in the blood and can even be used to power the TCA cycle in both normal tissue and tumors ^27,33^. It is known that tissues such as muscle are highly glycolytic and produce lactate that ends up in the circulation^35–37^. It is thought that lactate produced in the muscle then can act at a distance in a process known as the lactate shuttle, to participate in gluconeogenesis and even act as an agonist with the hormone receptor GRP81, such that lactate potentially acts as a “lactormone” ^27^. It is tempting to speculate that the lactate produced by tumors is meant to act as a signal to alert the entire body to the presence of a metabolic disruption, and therefore loss of lactate production in SCCs would not affect the progression of the tumor in our murine model, but instead how the entire system may respond to tumor formation.

Our original hypothesis based on the expression pattern and activity of *Ldha* during SCC progression was that Ldh inhibition would abrogate tumor growth. However, the data demonstrate instead that while in vivo deletion of *Ldha* did affect the metabolism of the tumors formed, this did not affect cancer cell proliferation, survival, pathology, immune response etc. Perhaps in vitro data showing that Ldh inhibition can block tumor growth are incomplete because tumor cells in vivo can take up lactate from the blood to make up for the loss of Ldh activity in the treated cells. Metabolomics data from SCCs without Ldh activity showed that whether tumors are making lactate or taking it up from the circulation, not only is the pool of lactate low in SCCs, but also lactate production is low. Furthermore, the lack of Ldh activity also corresponded to a negative feedback whereby all the glycolytic metabolites were decreased, suggesting that increased glucose utilization in general is not required for tumor initiation or progression in SCC. These results could provide a simple explanation for why several efforts to exploit Ldh inhibition to treat cancer have not progressed beyond early stage clinical trials.

Recent studies have suggested that tumors are metabolically flexible, which could explain why loss of Ldh activity did not affect initiation or progression of SCCs. In this scenario, tumors lacking the ability to use glucose to produce lactate simply take up other metabolites, such as glutamine to generate products necessary for increased biomass during proliferation. In response to loss of glucose catabolism, oxidative and reductive glutamine metabolism could be simultaneously increased across a tumor mass as a result of heterogeneity of oxygen tension across the tumor. Hypoxia is known to promote tumor reductive glutamine metabolism^38^, so it is conceivable that hypoxic regions could exhibit increased reductive glutamine metabolism whereas better perfused regions could exhibit enhance oxidation glutamine metabolism.

We used glutamine labeling to trace uptake and metabolism and did indeed find that *Ldha*-null tumors took up and used more glutamine to power their metabolism. Although there was no difference in [U-13C3] lactate labeling of TCA cycle metabolites, *Ldha*-null tumors increased uptake and TCA cycle metabolism of [U-13C5] glutamine, suggesting the use of glutamine as a carbon source to compensate for reduced glycolysis/glucose metabolism. These results suggest that *Ldha*-null tumors may be sensitized to glutaminase inhibition^35^. An outstanding question from this work is to understand whether this increased glutamine uptake and utilization compensates for loss of glucose metabolism in the absence of Ldh activity. It is possible that dual inhibition of both Ldh activity and glutamine uptake or glutaminase could potentially starve tumors by circumventing their metabolic flexibility, and this will be the focus of effort going forward.

## Methods

### Animal experiments

All animal experiments and related procedures were performed in accordance with protocols approved by the Institutional Animal Care and Use Committee (IACUC) at UCLA in facilities run by the UCLA Department of Laboratory Animal Medicine (DLAM). Animal strains came from Jackson Labs (*K15-CrePR, Lgr5-CreER*), the National Cancer Institute Mouse Models of Human Cancers Consortium repository (LSL-*Kras*G12D and *p53*fl/fl), the Rutter (Mpcfl/fl) and Seth laboratories (*Ldha*fl/fl) and were maintained under conditions set forth by IACUC and UCLA Animal Resource Committee. For tumor initiation experiments, *K15-CrePR* animals were shaved and treated by injection of mifepristone, and *Lgr5-CreER* animals were shaved and treated with tamoxifen (200 μl of 10 mg ml^−1^ dissolved in filtered sunflower seed oil daily for 3 days) during telogen (7–8 weeks postnatal), and monitored for hair and tumor growth following shaving. Tumors generated in *K15-CrePR* animals were harvested for analysis 8–9 weeks post mifepristone induction, and tumors generated in *Lgr5-CreER* animals were harvested 14–16 weeks post tamoxifen induction. For tumor progression experiments, tumors were generated in *K15-CrePR* and *Lgr5-CreER* animals floxed for either *Ldha* or *Mpc1* by cutaneous two-stage chemical carcinogenesis^39^. Briefly, transgenic animals (7–8 weeks postnatal) were shaved and treated once on the shaved dorsal skin with 200 nmol of DMBA dissolved in acetone. One week later, 5 nmol TPA dissolved in 100% ethanol was applied to the dorsal skin. In total, 5 nmol TPA treatment continued twice a week for the duration of the experiment. At the first visible sign of tumor formation, *K15-CrePR* and *Lgr5-CreER* mice were treated with mifepristone and tamoxifen, respectively to delete *Ldha* or *Mpc1*. Mifepristone or tamoxifen were administered by intraperitoneal injection (200 μl of 10 mg ml^−1^ dissolved in filtered sunflower seed oil daily for 3 days). Tumors were harvested for analysis 19–20 weeks post initial DMBA treatment. Both male and female animals were used in this study in approximately equal numbers with no apparent difference in phenotype between genders. All animals shown were maintained on a mixed C57BL6/FVB background. No statistical measure was used to determine the sample size beforehand. The results described include data from all treated animals. The investigators were not blinded to allocation during the experimental data collection, nor were the experiments randomized. The results shown are representative images from at least three independently treated animals per genotype as denoted in each experimental legend, and genotyping was performed both before and after animal treatment for confirmation.

### Histology and immunostaining

Tumors were isolated from animals of indicated genotypes and embedded fresh in OCT compound for frozen tissue preparations, or fixed overnight in 4% formalin and embedded in paraffin. Formalin-fixed paraffin-embedded (FFPE) tumor sections were cut at 5 μm and fresh frozen tumors in OCT compound were cut at 10 μm for hematoxylin and eosin staining, and immunostaining. Immunohistochemistry on FFPE tissue sections was performed (White et al.^15^). Briefly, paraffin-embedded tumor sections were de-paraffinized, rehydrated, and blocked in staining buffer containing appropriate control IgG (goat, rabbit etc.). Antigen retrieval was performed on formalin-fixed paraffin-embedded tumor sections with citrate or Tris-EDTA buffers for 30 min at 95 °C with the following antibodies: Ki-67 (Abcam, ab16667, 1:50), p-S6 (Cell Signaling, CST2215, 1:50), Sox9 (Abcam, ab185230, 1:1,000), CD34 (Abcam ab81289, 1:1000), K14 (Covance, PRB-155P, 1:800), Fibronectin (Abcam, ab2413, 1:250), Tenascin C (Abcam ab108930, 1:500). The DAKO EnVision + HRP Peroxidase System (Dako K400911-2) and Dako AEC Substrate Chromogen (Dako K346430-2) was used for detection. Images were collected on an Olympus BX43 Upright Microscope. For hypoxia staining, Hypoxyprobe^TM^-1 solution (pimonidazole HCl) (Hypoxyprobe, HP3-100Kit) was administered to mice by intraperitoneal injection at a dosage of 60 mg/kg 45 min before harvest of skin. Tissue was embedded, sectioned, and stained as described above using affinity purified anti-pimonidazole rabbit antisera containing 0.09% sodium azide and 1% BSA (PAb2627AP, 1:100).

### Western blotting

Fresh tumor samples were homogenized with a tissue microgrinder followed by mechanical dissociation with a syringe and cell lysis in RIPA buffer (Pierce) with Halt protease and phosphatase inhibitors (Thermo-Fisher) on ice. After removing insoluble material by centrifugation at 8000*g* at 4 °C for 5 min, total protein concentration was determined using the BCA assay kit (Pierce) per manufacturer’s protocol with a microplate reader. Twenty micrograms of protein per tumor sample was diluted in SDS-PAGE gel electrophoresis sample buffer (Bio-Rad) and boiled at 95 °C for 5 min. Denatured proteins were resolved on SDS-PAGE gels (NuPAGE Novex Gels, Thermo-Fisher) and transferred onto PVDF membranes (Bio-Rad). Blocking was done with 5% milk in PBST, and then membranes were incubated with primary antibodies; β-actin (Abcam, ab8227; 1:1000), Ldha (Cell Signaling, CST2012; 1:1000), *Mpc1* (Sigma HPA045119; 1:500) overnight at 4 °C. After washing, membranes were incubated with peroxidase-conjugated secondary antibodies for 1 h at room temperature. Signals were detected with Pierce ECL Western Blotting Substrate following washes.

### Ldh activity assay in tumor lysate

Ldh activity was determined in tumor cell lysates by measuring the formation of soluble XTT formazan in direct relation to production of NADH over time at 475 nm at 37 °C using a SynergyMX plate reader (Biotek Instruments). Fresh tumor samples were homogenized with a tissue microgrinder followed by mechanical dissociation with a syringe and cell lysis in RIPA buffer (Pierce) with Halt protease and phosphatase inhibitors (Thermo-Fisher) on ice. After removing insoluble material by centrifugation at 8000*g* at 4 °C for 5 min, total protein concentration was determined using the BCA assay kit (Pierce) per manufacturer’s protocol with a microplate reader. Ten micrograms of protein was used per well for each tumor. Samples were run in triplicates. The staining solution contained 50 mM Tris buffer pH 7.4, 150 μM XTT (Sigma), 750 μM NAD (Sigma), 80 μM phenazine methosulfate (Sigma), and 10 mM of substrate lactate (Sigma). Ldh activity was determined in cell lysates by measuring the change in absorbance of their common substrate or product, NADH, over time at 340 nm at 25 °C using a Synergy-MX plate reader (Biotek Instruments).

### In situ Ldh assay

Ten-micron cryostat sections of fresh frozen tumors were briefly fixed (4% formalin for 5 min), washed with PBS pH 7.4 for 10 min, and then incubated with the appropriate solution for Ldh activity. Ldh staining solution contained 50 mM Tris pH 7.4, 750 μM NAD (Sigma), 80 μM phenazine methosulfate (Sigma), 600 μM nitrotetrazolium blue chloride (Sigma), 10 mM MgCl_2_ (Sigma), and 10 mM of the substrate lactate (Sigma). Slides were incubated with staining solution at 37 °C until they reached the desired intensity, then counterstained using Nuclear Fast Red (Vector) or Braziliant! (Anatech) and mounted using VectaMount (Vector). Control reactions were performed by using staining solution that lacked the substrate mixture or NAD.

### Glutaminase activity assay

Glutaminase activity was determined in tumor cell lysates by measuring the formation of soluble XTT formazan in direct relation to production of NADH over time at 475 nm at 37 °C using a SynergyMX plate reader (Biotek Instruments). The glutaminase assay used was modified from the in situ assay described in Montero et al. and Botman et al. ^40,41^. Fresh tumor samples were homogenized with a tissue microgrinder followed by mechanical dissociation with a syringe and cell lysis in RIPA buffer (Pierce) with Halt protease and phosphatase inhibitors (Thermo-Fisher) on ice. After removing insoluble material by centrifugation at 8000*g* at 4 °C for 5 min, total protein concentration was determined using the BCA assay kit (Pierce) per manufacturer’s protocol with a microplate reader. Ten micrograms of protein was used per well for each tumor. Samples were run in triplicates. In control reactions either glutamine or phosphate was omitted, or the protein denatured. The staining solution contained 200 mM Tris-HCl buffer (pH 8.0); 200 mM KH_2_PO_4_; 40 mM glutamine; 0.1 mM EDTA; 3.4 mM NAD^+^ (Sigma); 0.5 mM ADP (Sigma); 0.3 mM XTT (Sigma); 0.49 mM phenazine methosulfate (Sigma); and 100 units of glutamate dehydrogenase used as an auxiliary enzyme (GIDH) (Sigma). Glutaminase activity was determined in cell lysates by measuring the change in absorbance of their common substrate or product, NADH, over time at 340 nm at 25 °C using a Synergy-MX plate reader (Biotek Instruments).

### LCMS-based metabolomics analysis

The experiments were performed as previously described^17^. To extract intracellular metabolites from tumor cells, fresh tumor samples of approximately the same volume (0.15 cm^3^) were briefly rinsed with cold 150 mM ammonium acetate (pH 7.3), followed by addition of 1 ml cold 80% methanol/20% water and homogenization on dry ice with a tissue microgrinder and mechanical dissociation through a syringe. Cell suspensions were transferred into Eppendorf tubes, and 10 nmol D/L-norvaline was added as a loading control for the instrument. After rigorously mixing, the suspension was pelleted by centrifugation (18,000*g*, 4 °C for 5 min). The supernatant was transferred into a glass vial, metabolites dried down under vacuum, and resuspended in 70% acetonitrile/30% water. Cell pellets were resuspended in RIPA buffer (Pierce) with Halt protease and phosphatase inhibitors (Thermo-Fisher) on ice. After removing insoluble material by centrifugation at 8000*g* at 4 °C for 5 min, total protein concentration was determined using the BCA assay kit (Pierce) per manufacturer’s protocol with a microplate reader. For the mass spectrometry-based analysis of the sample, 5 μl was injected onto a Luna NH2 (150 mm × 2 mm, Phenomenex) column. The samples were analyzed with an UltiMate 3000RSLC (Thermo Scientific) coupled to a Q Exactive mass spectrometer (Thermo Scientific). The Q Exactive was run with polarity switching (+3.50 kV/−3.50 kV) in full scan mode with an m/z range of 65–975. Separation was achieved using: (A) 5 mM NH4AcO (pH 9.9) and (B) ACN. The gradient started with 15% (A) going to 90% (A) over 18 min, followed by an isocratic step for 9 min and reversal to the initial 15% (A) for 7 min. Metabolites were identified with TraceFinder 3.3 using accurate mass measurements (≤3 ppm) and retention times. Metabolite data were normalized to tumor protein concentration and norvaline as an internal standard for metabolite extraction and are available at figshare.com (10.6084/m9.figshare.c.3801271). For experiments with labeled isotope tracing ([U-^13^C_6_] glucose, [U-^13^C_5_] glutamine, and [U-^13^C_3_] lactate; Cambridge Isotope Laboratories Inc. CLM-1396-5, CLM-1822-H-0.1, CLM-1579-0.5), the labeled isotope was delivered by intraperitoneal injection (2 g/kg in PBS) 15 min prior to euthanasia. Tumor samples were then harvested and prepared as described above.

### RNaseq and bioinformatics

To compare gene expression profiles from tumors with and without *Ldha*, total RNA was isolated from fresh tumor samples. Fresh tumor samples were placed in Trizol LS reagent and homogenized by vortexing, microgrinding, and mechanical dissociation through a syringe. Total RNA isolation was subsequently performed using an RNeasy Mini Kit (Qiagen) following the manufacturer’s protocol with chloroform, isopropanol, and ethanol washes.

Total RNA sample QC: All samples need to pass through the following four steps before library construction: (1) Nanodrop: tests RNA purity (OD260/OD280), (2) Agarose Gel Electrophoresis, (3) Agilent 2100 to check RNA integrity.

Library construction: After the QC procedures, mRNA from tumor samples was enriched using oligo(dT) beads. The mRNA was then fragmented randomly in fragmentation buffer, followed by cDNA synthesis using random hexamers and reverse transcriptase. After first-strand synthesis, a custom second-strand synthesis buffer (Illumina) was added with dNTPs, RNase H, and *Escherichia coli* polymerase I to generate the second strand by nick-translation. The final cDNA library was ready after a round of purification, terminal repair, A-tailing, ligation of sequencing adapters, size selection, and PCR enrichment.

Library QC: Library concentration was first quantified using a Qubit 2.0 fluorometer (Life Technologies), and then diluted to 1 ng/µl before checking insert size on an Agilent 2100 and quantifying to greater accuracy by quantitative PCR (Q-PCR) (library activity >2 nM).

Sequencing: Libraries were sequenced on HiSeq2500 (Illumina).

Data filtering: Raw reads were filtered to remove reads containing adapters or reads of low quality, so that downstream analyses were based on clean reads. The filtering process was as follows: (1) discard reads with adaptor contamination, (2) discard reads when uncertain nucleotides constitute more than 10% of either read (*N* > 10%), (3) discard reads when low quality nucleotides (base quality <20) constitute >50% of the read.

RNA-seq Adapter sequences (Oligonucleotide sequences of adapters from TruSeqTM RNA and DNA Sample Prep Kits):

RNA 5ʹ Adapter (RA5), part # 15013205:

5ʹ-AATGATACGGCGACCACCGAGATCTACACTCTTTCCCTACACGACGCTCTTCCGATCT-3ʹ

RNA 3ʹ Adapter (RA3), part # 15013207: 5ʹ-GATCGGAAGAGCACACGTCTGAACTCCAGTCAC (6-nucleotide index) ATCTCGTATGCCGTCTTCTGCTTG-3ʹ

Mapping to a reference genome: Algorithm for mapping sequences: appropriate software was chosen according to the characteristics of the reference genome. TopHat2 was run for tumor genomes. The mismatch parameter were set to two, and other parameters were set to default. Only filtered reads are used to analyze the mapping status of RNA-seq data to the reference genome.

Expression quantification: Gene expression level was measured by transcript abundance to generate FPKM counts, short for the expected number of Fragments Per Kilobase of transcript sequence per Millions base pairs sequenced, which takes into account the effects of both sequencing depth and gene length counting of fragments. HTSeq software was used to analyze the gene expression levels in this experiment, using the union mode. The result files present the number of genes with different expression levels and the expression level of single genes. In general, an FPKM value of 0.1 or 1 is set as the threshold for determining whether the gene is expressed or not. Fragments Per Kilobase of transcript per Million mapped reads (fkpm) values were ranked by the log_2_-transformed foldchange for knockout versus wild-type.

### FDG-PET imaging and analysis

Small-animal PET/CT scans were performed using microPET/CT system Genisys 8 (Sofie Bioscience). Mice were fasted for 4 h, placed on a heating pad to warm the mice for 60 min, and then anesthetized using 1.5–2% isoflurane. 20 μCi of ^18^F-FDG probes was administrated via tail vein. Acquisition of static PET images was started 60 min after probe injection. Maximum-likelihood expectation maximization with 60 iterations was used for PET image reconstruction. All images were corrected for photon attenuation. The CT acquisition parameters were 40 kVp, 190 mA, and 720 projections with an exposure time of 55 ms at each projection. ^86^Y-AABD PET imaging was acquired 14 h after injection. For image analysis, PET/CT images were analyzed using OsiriX Imaging Software (Version 3.9.3; Pixmeo SARL, Bernex, Switzerland).

### Statistics and reproducibility

Experiments were performed on male and female animals in approximately equal numbers with no apparent difference in phenotype between sexes. All phenotypes described are representative of a minimum of *n* = 3 littermate pairs (or a total of six mice) as indicated in the description of each experiment. For analysis of the hair regrowth phenotype, no statistical measure was used to determine the sample size beforehand, nor were statistics used to measure effects, as the results were essentially positive or negative as represented in the figures. The results described include data from all treated animals. Investigators were not blinded to allocation during the experimental data collection. Experiments were not randomized. All results shown are representative images from at least three independently treated animals, and genotyping was performed both before and after animal treatment for confirmation. Pairwise comparisons between two groups were performed by two-tailed statistical analysis using Student’s *t* test. Statistical significances were considered if *p < 0.05; **p < 0.01; ***p < 0.001. Experimental data are demonstrated as the mean ± SEM. Sample size and statistical details can be found in the figure legends.

## Supplementary information

Supplementary Information

## Data Availability

Previously published transcriptomics data that were reanalyzed here are available under accession (NIH GEO GSE111997). Normalized metabolite data are available at figshare.com: Figure 1 c, d; Fig. 3 d–f; Supplementary Figure 4 (Exp 5), 10.6084/m9.figshare.7194761 [https://figshare.com/s/4d9b59a0e4fd85dc9c9b]. Figure 1 c, d; Fig. 3d–f; Supplementary Figure 4 (Exp 4), 10.6084/m9.figshare.7194809 [https://figshare.com/s/df136b2a3154195e7315]. Figure 1c, d; Fig. 3d–f; Supplementary Figure 4 (Exp 3), 10.6084/m9.figshare.7194833 [https://figshare.com/s/86b1503620e50cdd5fe9]. Figure 1 c, d; Fig. 3d–f; Supplementary Figure 4 (Exp 2), 10.6084/m9.figshare.7194836 [https://figshare.com/s/f33a0d482412cef627e3]. Figure 1 c, d; Fig. 3d–f; Supplementary Figure 4 (Exp 1), 10.6084/m9.figshare.7194839 [https://figshare.com/s/34f29012ddec6e457e1b]. Supplementary Figure 5, 10.6084/m9.figshare.7194851 [https://figshare.com/s/c2f7dd6edfededce0239]. Figure 7a, d–f 10.6084/m9.figshare.7194857 [https://figshare.com/s/48038002f6d30ed28b28]. Figure 6g (Exp 2) 10.6084/m9.figshare.7194860 [https://figshare.com/s/2a245272e321f993d360]. Figure 6g (Exp 1) 10.6084/m9.figshare.7194869 [https://figshare.com/s/297a51614d6eecd09443]. All other data supporting the findings of this study are available from the corresponding author on reasonable request.

## References

[1] Paldino E, Tesori V, Casalbore P, Gasbarrini A, Puglisi MA (2014). Tumor Initiating Cells and Chemoresistance: Which Is the Best Strategy to Target Colon Cancer Stem Cells?. Biomed. Res. Int..

[2] Warburg O (1956). On respiratory impairment in cancer cells. Science.

[3] Warburg O (1956). On the origin of cancer cells. Science.

[4] Palsson-McDermott EM, O'Neill LA (2013). The Warburg effect then and now: from cancer to inflammatory diseases. Bioessays.

[5] Bensinger SJ, Christofk HR (2012). New aspects of the Warburg effect in cancer cell biology. Semin. Cell. Dev. Biol..

[6] Vander Heiden MG, Cantley LC, Thompson CB (2009). Understanding the Warburg effect: the metabolic requirements of cell proliferation. Science.

[7] Doherty JR, Cleveland JL (2013). Targeting lactate metabolism for cancer therapeutics. J. Clin. Invest..

[8] Husain Z, Huang Y, Seth P, Sukhatme VP (2013). Tumor-derived lactate modifies antitumor immune response: effect on myeloid-derived suppressor cells and NK cells. J. Immunol..

[9] Seth P (2011). On-target inhibition of tumor fermentative glycolysis as visualized by hyperpolarized pyruvate. Neoplasia.

[10] Xie H (2009). LDH-A inhibition, a therapeutic strategy for treatment of hereditary leiomyomatosis and renal cell cancer. Mol. Cancer Ther..

[11] Xie H (2014). Targeting lactate dehydrogenase--a inhibits tumorigenesis and tumor progression in mouse models of lung cancer and impacts tumor-initiating cells. Cell. Metab..

[12] Shim H (1997). c-Myc transactivation of LDH-A: implications for tumor metabolism and growth. Proc. Natl. Acad. Sci. USA.

[13] Le A (2010). Inhibition of lactate dehydrogenase A induces oxidative stress and inhibits tumor progression. Proc. Natl. Acad. Sci. USA.

[14] Fantin VR, St-Pierre J, Leder P (2006). Attenuation of LDH-A expression uncovers a link between glycolysis, mitochondrial physiology, and tumor maintenance. Cancer Cell..

[15] White AC (2011). Defining the origins of Ras/p53-mediated squamous cell carcinoma. Proc. Natl. Acad. Sci. USA.

[16] Lapouge G (2011). Identifying the cellular origin of squamous skin tumors. Proc. Natl. Acad. Sci. USA.

[17] Flores A (2017). Lactate dehydrogenase activity drives hair follicle stem cell activation. Nat. Cell Biol..

[18] Leushacke M, Barker N (2012). Lgr5 and Lgr6 as markers to study adult stem cell roles in selfrenewal and cancer. Oncogene.

[19] Jaks V (2008). Lgr5 marks cycling, yet long-lived, hair follicle stem cells. Nat. Genet..

[20] White AC (2014). Stem cell quiescence acts as a tumour suppressor in squamous tumours. Nat. Cell Biol..

[21] Girgis H (2014). Lactate dehydrogenase A is a potential prognostic marker in clear cell renal cell carcinoma. Mol. Cancer.

[22] Blanpain C, Lowry WE, Geoghegan A, Polak L, Fuchs E (2004). Self-renewal, multipotency, and the existence of two cell populations within an epithelial stem cell niche. Cell.

[23] Morris RJ (2004). Capturing and profiling adult hair follicle stem cells. Nat. Biotechnol..

[24] Trempus CS (2003). Enrichment for living murine keratinocytes from the hair follicle bulge with the cell surface marker CD34. J. Invest. Dermatol..

[25] Wang J (2013). Fast metabolic response to drug intervention through analysis on a miniaturized, highly integrated molecular imaging system. J. Nucl. Med..

[26] Palaskas N (2011). 18F-fluorodeoxy-glucose positron emission tomography marks MYCoverexpressing human basal-like breast cancers. Cancer Res..

[27] San-Millan I, Brooks GA (2017). Reexamining cancer metabolism: lactate production for carcinogenesis could be the purpose and explanation of the Warburg Effect. Carcinogenesis.

[28] Pavlova NN, Thompson CB (2016). The emerging hallmarks of cancer metabolism. Cell. Metab..

[29] Vander Heiden MG, DeBerardinis RJ (2017). Understanding the intersections between metabolism and cancer biology. Cell.

[30] Thonsri U (2017). Overexpression of lactate dehydrogenase A in cholangiocarcinoma is correlated with poor prognosis. Histol. Histopathol..

[31] Huang X (2016). High expressions of LDHA and AMPK as prognostic biomarkers for breast cancer. Breast.

[32] Cortazar AR (2018). CANCERTOOL: a visualization and representation interface to exploit cancer datasets. Cancer Res..

[33] Schweizer J, Loehrke H, Hesse B, Goerttler K (1982). 7,12-Dimethylbenz[a]anthracene/12-O-tetradecanoyl-phorbol-13-acetate-mediated skin tumor initiation and promotion in male Sprague-Dawley rats. Carcinogenesis.

[34] Schell JC (2017). Control of intestinal stem cell function and proliferation by mitochondrial pyruvate metabolism. Nat. Cell Biol..

[35] Momcilovic M (2018). The GSK3 signaling axis regulates adaptive glutamine metabolism in lung squamous cell carcinoma. Cancer Cell..

[36] Hui S (2017). Glucose feeds the TCA cycle via circulating lactate. Nature.

[37] Faubert B (2017). Lactate metabolism in human lung tumors. Cell.

[38] Metallo CM (2011). Reductive glutamine metabolism by IDH1 mediates lipogenesis under hypoxia. Nature.

[39] Filler, R. B., Roberts, S. J., Girardi, M. Cutaneous two-stage chemical carcinogenesis. *Cold Spring Harbor Protocols* 10.1101/pdb.prot4837 (2007) 10.1101/pdb.prot4837 21357170

[40] Montero F (2007). Glutaminase activity is confined to the mantle of the islets of Langerhans. Biochimie.

[41] Botman D (2014). Determination of phosphate-activated glutaminase activity and its kinetics in mouse tissues using metabolic mapping (quantitative enzyme histochemistry). J. Histochem. Cytochem..

