# Author Correction: Increased lactate dehydrogenase activity is dispensable in squamous carcinoma cells of origin

**DOI:** 10.1038/s41467-019-09435-z

**Published:** 2019-03-26

**Authors:** A. Flores, S. Sandoval-Gonzalez, R. Takahashi, A. Krall, L. Sathe, L. Wei, C. Radu, J. H. Joly, N. A. Graham, H. R. Christofk, W. E. Lowry

**Affiliations:** 1 Department of Molecular Cell and Developmental Biology, UCLA, Los Angeles, 90095 CA USA; 2 Broad Center for Regenerative Medicine, UCLA, Los Angeles, 90095 CA USA; 3 Division of Dermatology, David Geffen School of Medicine, UCLA, Los Angeles, 90095 CA USA; 4 Department of Biological Chemistry, UCLA, Los Angeles, 90095 CA USA; 5 Department of Pharmacology, UCLA, Los Angeles, 90095 CA USA; 6 Department of Engineering, USC, Los Angeles, 90089 CA USA; 7 Mork Family Department of Chemical Engineering and Materials Science, University of Southern California, Los Angeles, 90089 CA USA; 8 Norris Comprehensive Cancer Center, University of Southern California, Los Angeles, 90089 CA USA; 9 Molecular Biology Institute, UCLA, Los Angeles, 90095 CA USA; 10 Jonsson Comprehensive Cancer Center, UCLA, Los Angeles, 90095 CA USA

Correction to: *Nature Communications* 10.1038/s41467-018-07857-9, published online 09 January 2019

The original version of this Article contained an error in the spelling of the authors J. H. Joly and N. A. Graham, which were incorrectly given as J. Jolly and N. Graham.

Additionally, the affiliation of both authors with ‘Mork Family Department of Chemical Engineering and Materials Science, University of Southern California, Los Angeles, CA 90089’ and N. A. Graham with 'Norris Comprehensive Cancer Center, University of Southern California, Los Angeles, CA 90089' was inadvertently omitted.

This has now been corrected in both the PDF and HTML versions of the Article.

