# Supplementary material for: Increased lactate dehydrogenase activity is dispensable in squamous carcinoma cells of origin
Source: Nat Commun. 2019 Jan 9;10:91. doi: 10.1038/s41467-018-07857-9 (PMC6327029; doi:10.1038/s41467-018-07857-9)
Supplement: Supplementary file 1 — Supplementary Information [file 41467_2018_7857_MOESM1_ESM.docx]

**
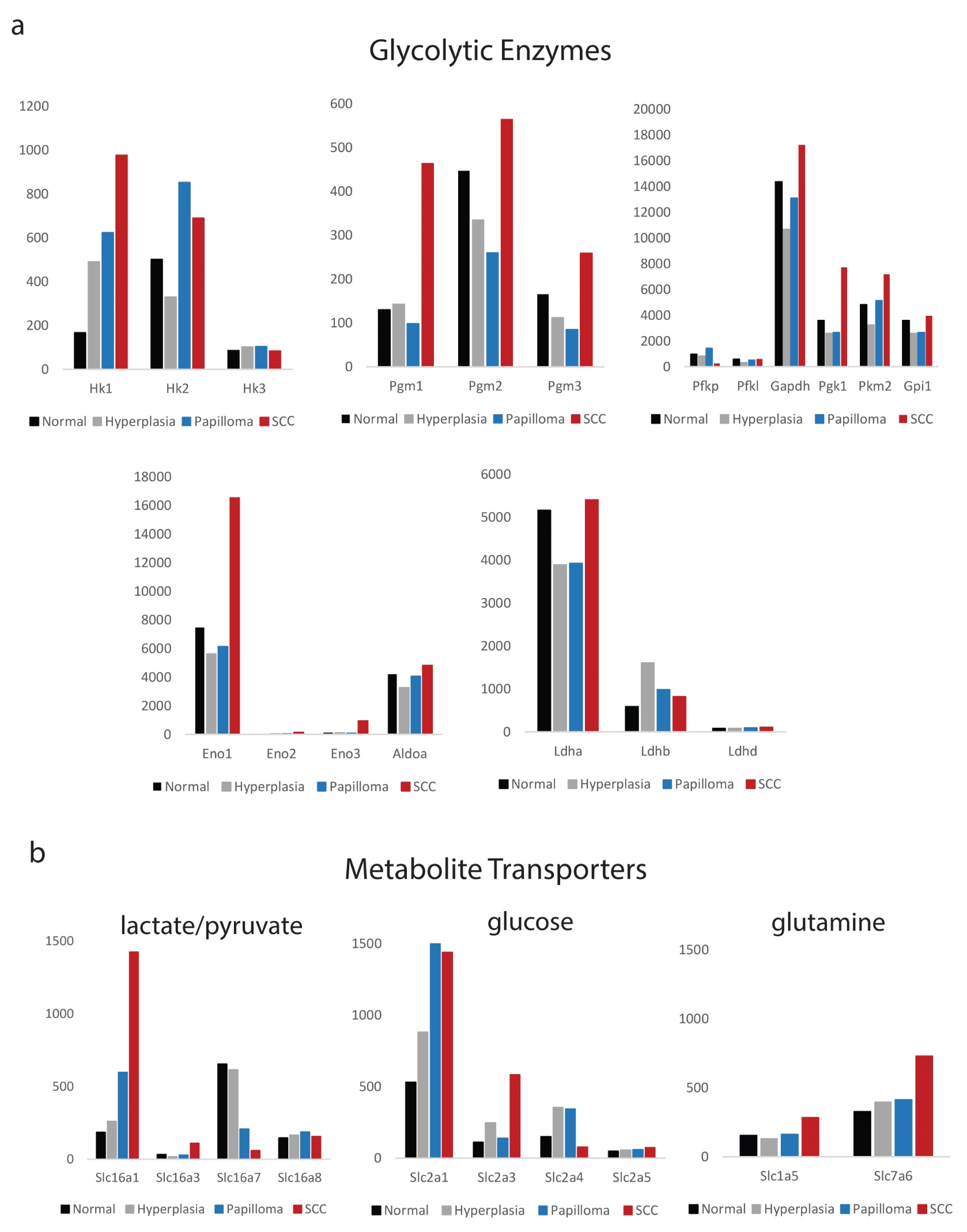
**

**Supplementary Figure 1. Transcriptome changes during SCC initiation and progression.**

Transcriptome data from a previous study on HFSC-driven SCC shows a dynamic pattern of expression of glycolytic enzymes over varying stages of tumorigenesis. The data presented are from cells labeled at the HFSC stage and then purified during the indicated stages of tumorigenesis. While *Ldha* was high in HFSCs and remained high at all stages of tumorigenesis, *Pgm, Hk, Pgk, Pkm, Eno* were all induced across tumorigenesis.

**
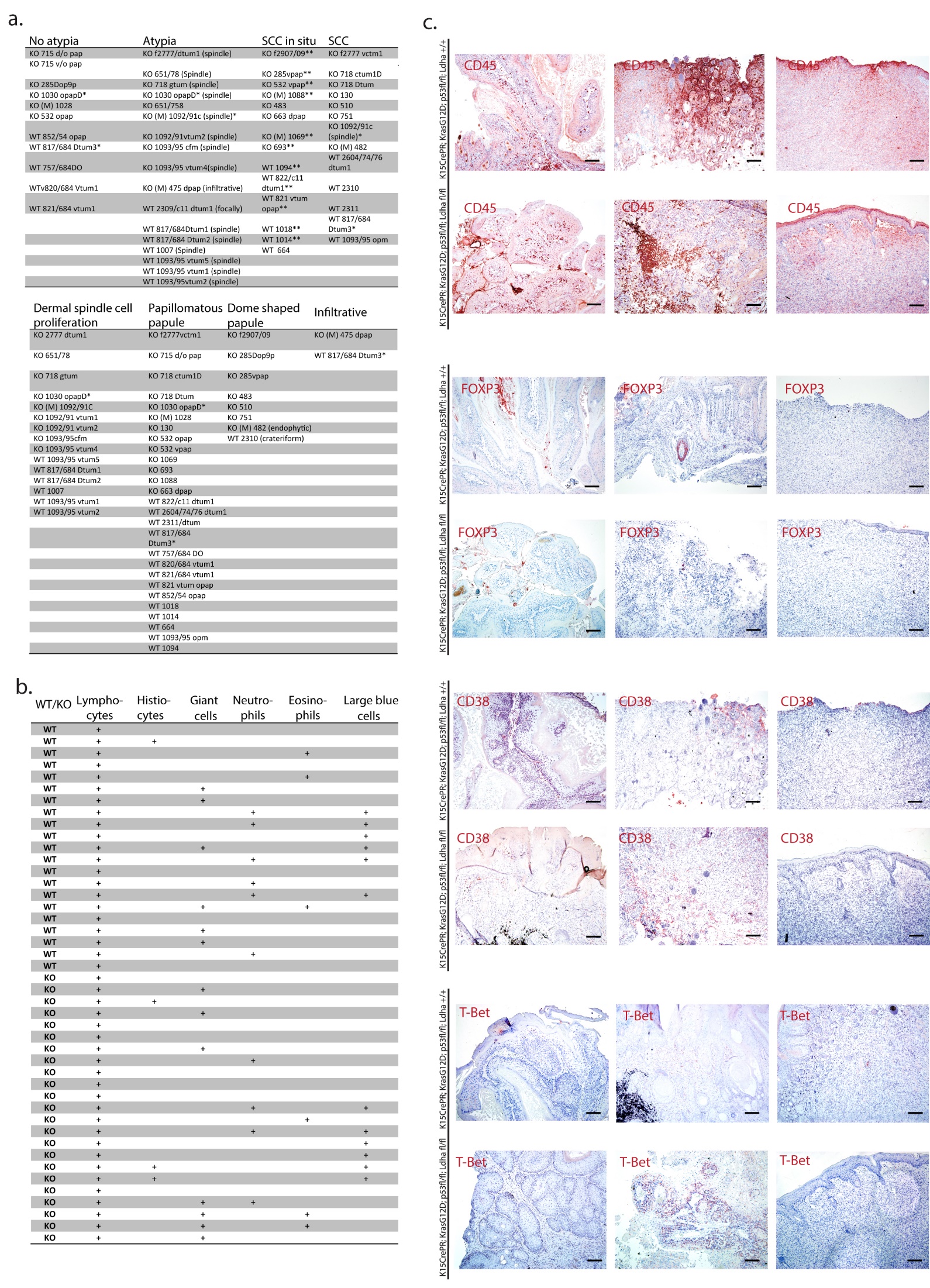
**

**Supplementary Figure 2, Pathological examination of tumorigenesis in the absence of Ldha. a**, Pathological examination of tumors generated with or without *Ldha* showed no differences in appearance, features, atypia etc. **b**, an examination of immune response to tumor formation showed a variety of immune features in SCC progression, but no difference based on expression of *Ldha*. **c**, Immunostaining for markers of T-cell subtypes in tumors with and without *Ldha*. Scale bars, 100µm.


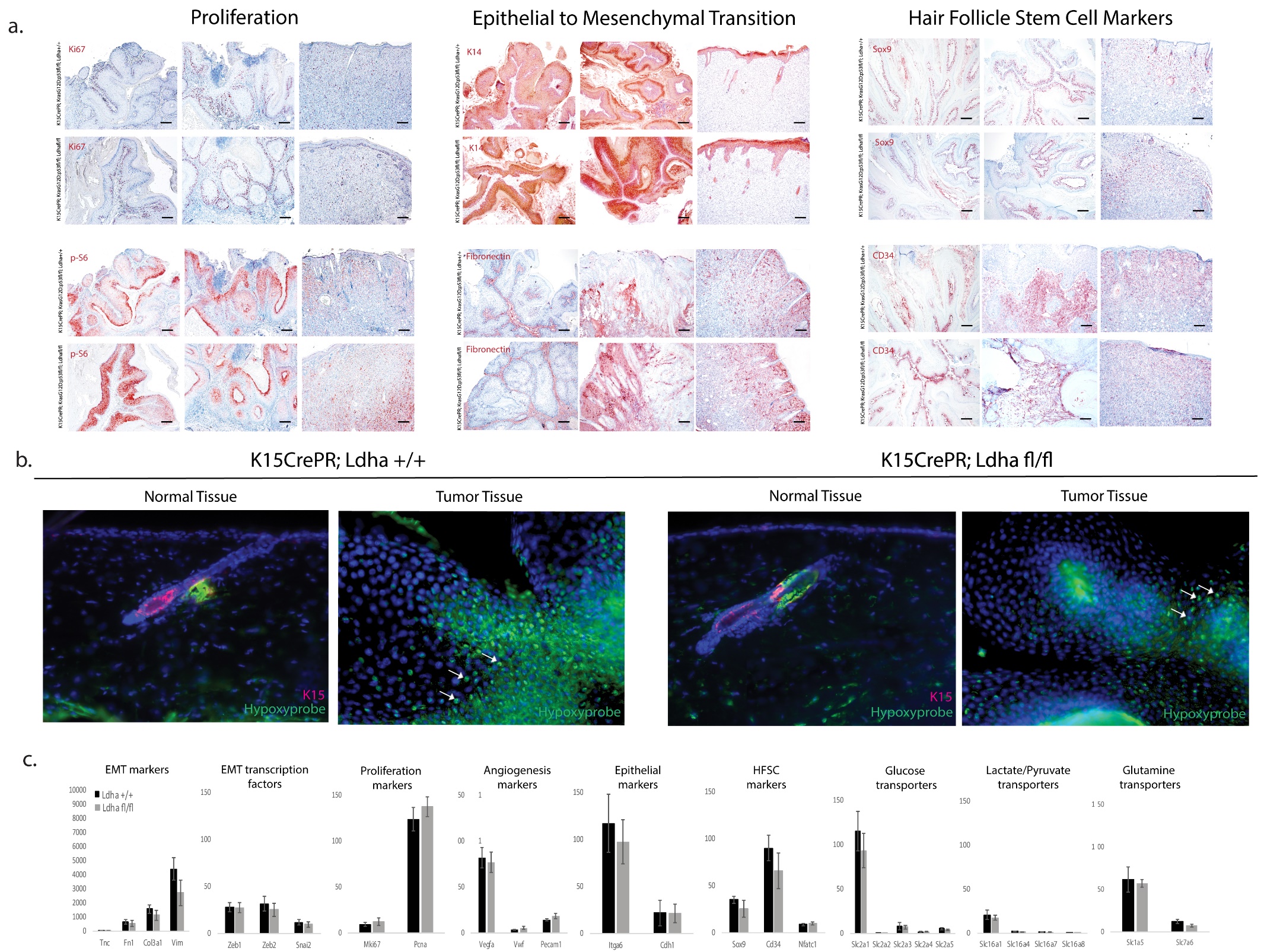


**Supplementary Figure 3. Extended characterization of SCCs generated with and without Ldha a**, Proliferating cells in the tumor were highlighted by immunostaining with an antibody against Ki67. **middle panel,** Evidence for Epithelial to Mesenchymal Transition (EMT) was assessed by immunostaining for Keratin 14, an epithelial marker and Fibronectin, a mesenchymal marker. **right panel**, To measure the expansion of HFSC fate in the tumors formed, we immunostained for markers Cd34 and Sox9. Scale bars, 100µm. **b**, Immunofluorescence staining for hypoxia levels in control versus *Ldha*-null tissue and tumors. White arrows indicate tumor cells as positive control for Hypoxyprobe. Scale bars, 50µm. **c**, RNA-seq on tumors with and without Ldh activity failed to uncover significantly expressed genes (Summarized in Supplementary Table 1 and 2), and no significant difference in the genes presented in Supplementary Figure 1.

**
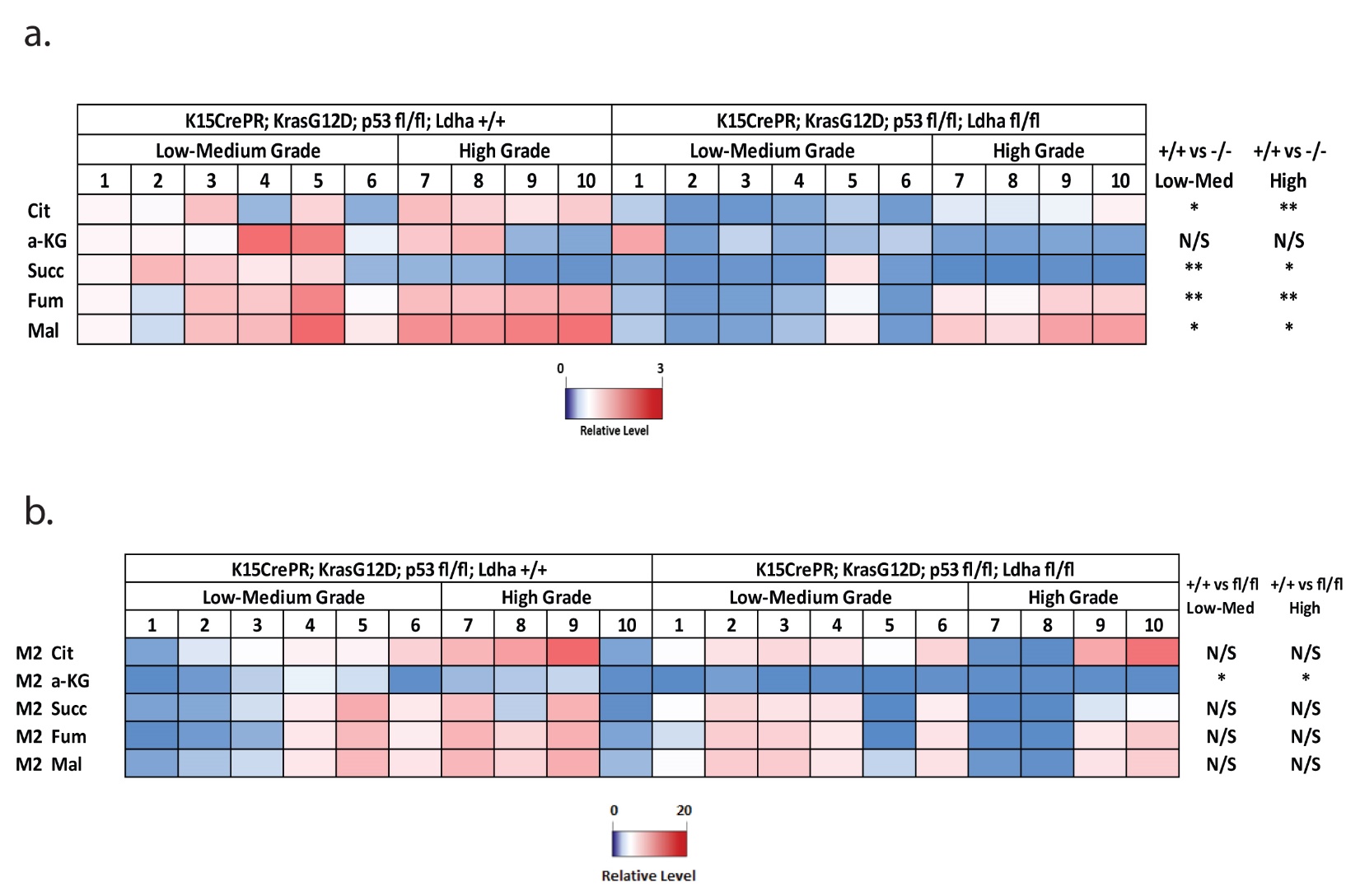
**

**Supplementary Figure 4. Examination of TCA metabolites in tumors** **a,** Heatmap depicts relative levels of TCA cycle intermediates as measured by LCMS in tumors from animals with and without *Ldha* expression. Each column represents metabolite measurements from an individual animal, and 20 animals were used, 10 of each genotype. **b,** Heatmap depicts percentage of TCA cycle intermediate isotopomers in tumors with indicated genotypes from animals IP injected with [U-^13^C_6_] glucose 15 minutes prior to tumor harvesting. Student’s paired t-test was performed, ∗P < 0.05; ∗∗P < 0.01; ∗∗∗P < 0.001; NS, not significant; n = 12.


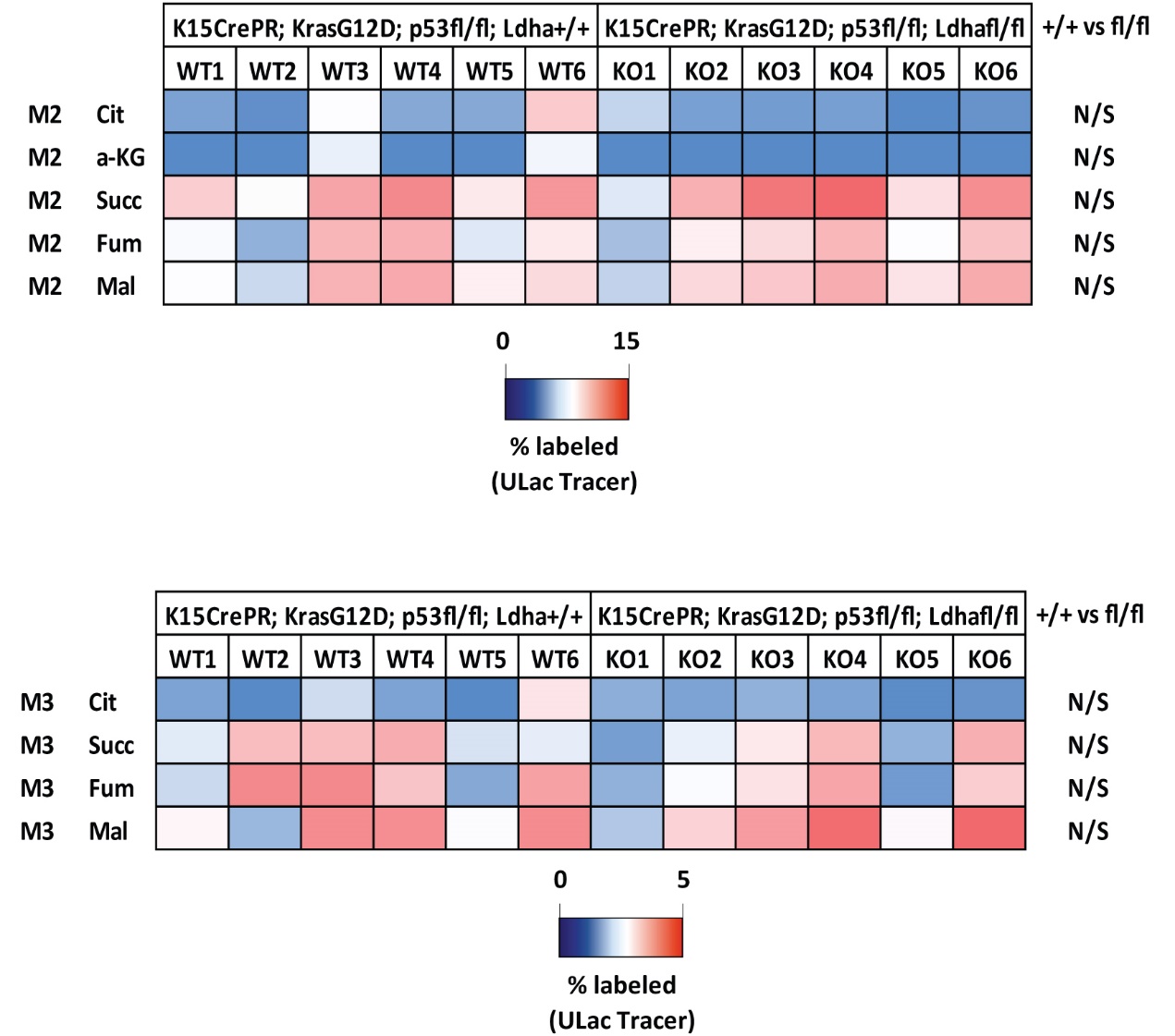


**Supplementary Figure 5. Lactate contribution to TCA cycle carbon is unaltered in Ldha-null SCC tumors.** Heatmap depicts percentage of TCA cycle intermediate isotopomers in tumors with indicated genotypes from animals IP injected with [U-^13^C_3_] lactate 15 minutes prior to tumor harvesting and metabolite extraction. Student’s paired t-test was performed, ∗*p*< 0.05; ∗∗*p* < 0.01; ∗∗∗*p* < 0.001; NS, not significant; n = 12.

**
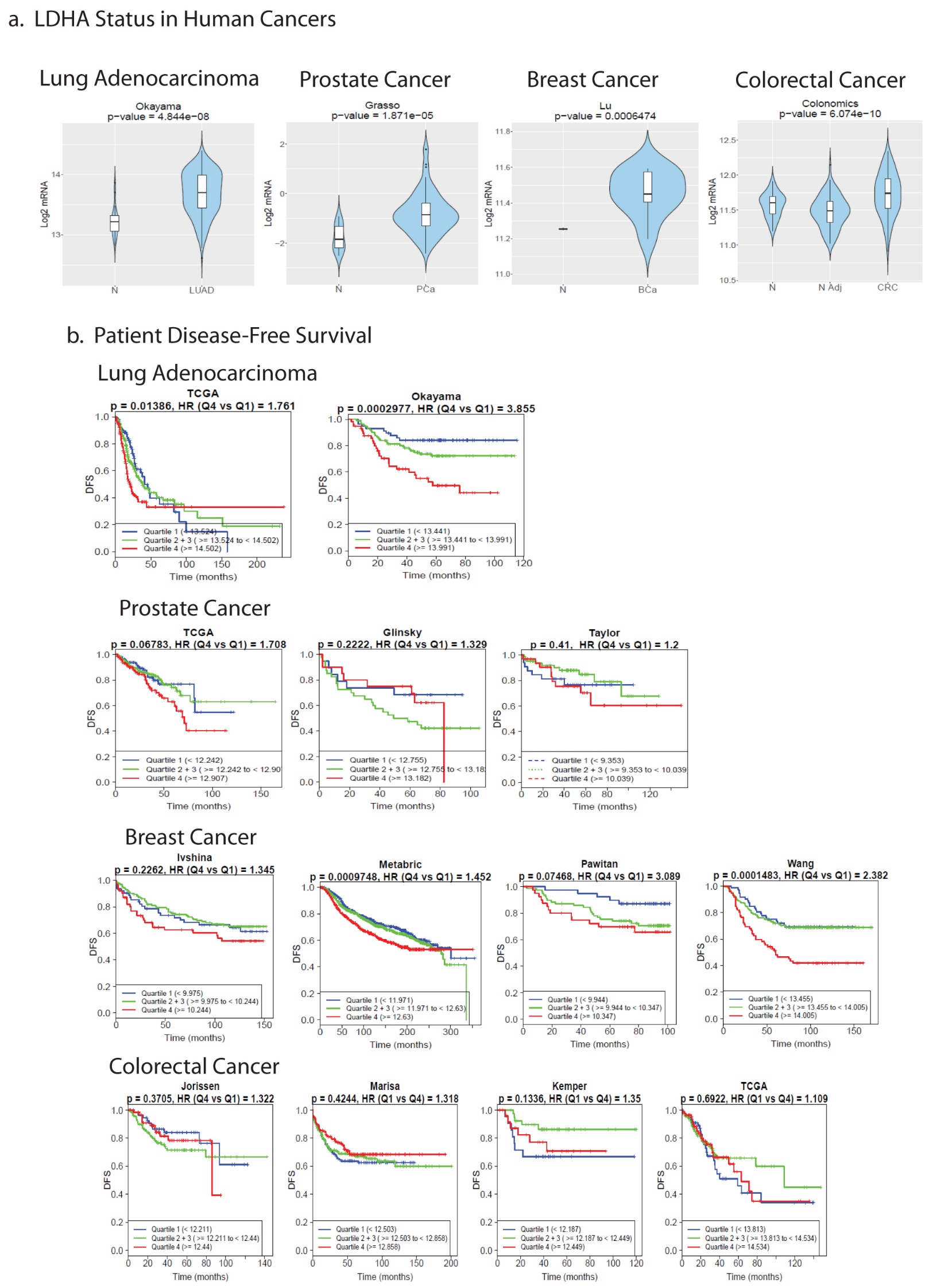
**

**Supplementary Figure 6. CANCERTOOL analysis to explore LDHA in Human Cancers a.** Violin plots depicting the expression of the gene of interest between non-tumoral (N) and cancer specimens (LUAD, PCa, BCa, CRC) in the indicated datasets. The Y-axis represents theLog2-normalized gene expression (fluorescence intensity values for microarray data or, sequencing reads values obtained after gene quantification with RSEM and normalization using Upper Quartile in case of RNAseq). A Student T-test is performed in order to compare the mean gene expression between two groups. **b.** Kaplan-Meier curves representing the disease-free survival (DFS) of patient groups selected according to the quartile expression of the gene of interest. Quartiles represent ranges of expression that divide the set of values into quarters. Quartile color code: Q1 (Blue), Q2 plus Q3 (Green), Q4 (Red). Each curve represents the percentage (Y-axis) of the population that exhibits recurrence of the disease along time (X-axis, in months) for a given gene expression distribution quartile. Vertical ticks indicate censored patients. Quartile colour code: Q1 (Blue), Q2 plus Q3 (Green), Q4 (Red). A Mantel-Cox test is performed in order to compare the differences between curves, while a Cox proportional hazards regression model is performed to calculate de Hazard Ratio (HR) between the indicated groups. The analyses in **a** and **b** were performed in CANCERTOOL^41^.
